# The immune landscape of IgA induction in the gut

**DOI:** 10.1007/s00281-021-00879-4

**Published:** 2021-08-11

**Authors:** Claudia Seikrit, Oliver Pabst

**Affiliations:** 1 Division of Nephrology and Clinical Immunology, RWTH Aachen University, Aachen, Germany; 2 Institute of Molecular Medicine, RWTH Aachen University, Pauwelsstr. 30, 52074 Aachen, Germany

**Keywords:** IgA biology, IgA nephropathy, Secretory IgA, Mucosal immune system

## Abstract

Antibodies are key elements of protective immunity. In the mucosal immune system in particular, secretory immunoglobulin A (SIgA), the most abundantly produced antibody isotype, protects against infections, shields the mucosal surface from toxins and environmental factors, and regulates immune homeostasis and a peaceful coexistence with our microbiota. However, the dark side of IgA biology promotes the formation of immune complexes and provokes pathologies, e.g., IgA nephropathy (IgAN). The precise mechanisms of how IgA responses become deregulated and pathogenic in IgAN remain unresolved. Yet, as the field of microbiota research moved into the limelight, our basic understanding of IgA biology has been taking a leap forward. Here, we discuss the structure of IgA, the anatomical and cellular foundation of mucosal antibody responses, and current concepts of how we envision the interaction of SIgA and the microbiota. We center on key concepts in the field while taking account of both historic findings and exciting new observations to provide a comprehensive groundwork for the understanding of IgA biology from the perspective of a mucosal immunologist.

## The many lives of IgA

In 1963, immunoglobulin (Ig) A was reported as a major component in mucosal secretions such as tears, bile, saliva, colostrum, and intestinal secretions [1, 2]. Another landmark paper in 1984 provided direct evidence to show how IgA is transported by the polymeric Ig receptor (pIgR) across mucosal epithelia [3] and exciting work followed to discover pathways of IgA induction and its potential for oral vaccination. Much of this work emphasized important differences between mucosal immunity and IgA on the one hand and systemic immune responses including IgG on the other hand. The idea of a distinct mucosal immune system was born.

In nephrology, a particular interest in IgA developed after Jean Berger described IgA nephropathy (IgAN) in 1968 [4]. However, rather than moving forward together, IgA-centered research has followed mostly independent pathways in mucosal immunology and nephrology. We propose that stronger cooperation between both fields will help to cast a more comprehensive picture of IgAN. While this review is aimed at an audience of nephrologists, we will approach the topic of IgA from the perspective of mucosal immunologists. We will structure this review along key concepts in IgA biology and highlight controversies which dominate the conversation in the mucosal immunology community. Some of these topics have immediate relevance for IgAN pathogenesis whereas other aspects might seem less directly relevant in the context of IgAN. Nevertheless, we anticipate that mutual understanding of the approaches and models prevalent in the two fields will enable the advent of a joint IgA research community. With that aim in mind, here, we provide a comprehensive and consistent framework of IgA biology in both mucosal tissues and peripheral organs, including the kidney.

## The structure of the IgA molecule

**Fig. 1:**
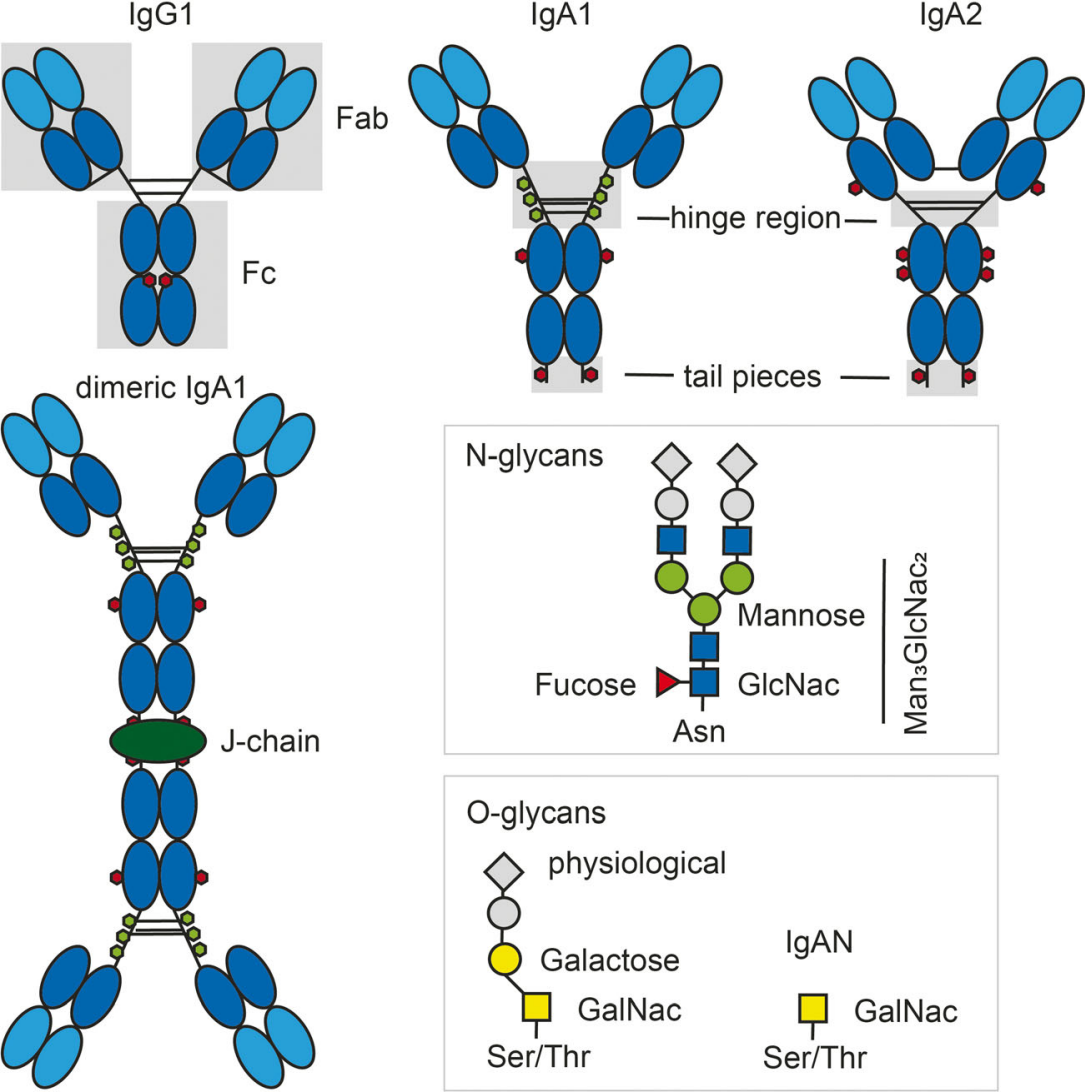
Structure of selected human Igs. The diagrams depict human IgG1, monomeric IgA1 and IgA2, and dimeric IgA1. The gray boxes indicate key domains of the respective molecules. Fab arms and the Fc part are depicted for IgG1; the hinge regions and tailpieces are indicated for IgA1 and IgA2. Positioning of O- and N-glycans are indicated by green- and red-filled hexagons respectively. In dimeric IgA, the tailpiece cysteines of two monomeric IgA molecules are covalently linked to the joining (J) chain depicted by green-filled ellipsoid. Disulfide bridges linking different proteins are displayed as lines. In secretory IgA, an additional molecule named secretory component is covalently bound to the complex (not depicted). The major differences between IgA1 and IgA2 are within the hinge region linking the Fc part and Fab part of the antibody. In IgA1, the hinge region is longer as compared to IgA2 and decorated by 3-6 O-linked oligosaccharides (3 depicted) [5]. N-Glycans show the common core of Man_3_GlcNac_2_ residues linked to asparagine (Asn). Complex N-glycans can have several branches each initiated by GlcNac (N-acetylglucosamine) and build complex and variable structures (represented by gray-filled symbols). Few studies have reported changes of N-glycans in IgAN patients as compared to healthy controls. O-Glycans consist of serine (Ser)- or threonine (Thr)-linked N-acetylgalactosamine (GalNac) with β1-3-linked galactose and variable sialylation. O-Glycans are typically less branched than N-linked glycans and show characteristic glycan truncation in IgAN patients as compared to healthy controls. The illustration depicts glycan core structures and characteristic changes in IgAN. However, glycan structures exhibit a wide heterogeneity and the exact structure of IgA-decorating glycans varies (figure adapted from [6–8])

IgA is secreted by class-switched plasma cells and shows that basic core structure common to all human Igs. Two identical heavy chains are covalently linked to two identical light chains, with each heavy/light chain pair forming a specific antigen-binding Fab arm of the antibody (Fig. 1). In humans (but not in mice), two IgA isotypes are present, IgA1 and IgA2. IgA1 and IgA2 are differentially represented in different compartments and have distinct properties. For example, in blood, the ratio of IgA1 to IgA2 is 10:1, whereas the ratio is 3:2 in the middle small intestine, and about equal proportions of IgA1 and IgA2 are detected in the colon [9]. IgA1 and IgA2 are generated by class switch recombination. Thus, a plasma cell can secrete either IgA1 or IgA2 at one time (see Box 1, caveats in understanding IgA). A major structural difference between IgA1 and IgA2 is found in their hinge region, a short stretch of less structured amino acids linking the Fab and Fc parts of the antibody (Fig. 1). In IgA2, this hinge region is 16 amino acids shorter than in IgA1 and lacks O-linked glycans [6]. The shorter length of the hinge region reduces the susceptibility of IgA2 for proteolytic cleavage [10] and thereby may increase IgA2 stability in the microbiota dense and highly proteolytic environment of the colon.

**Box 1:** Caveats in understanding human gut IgA biology

Structural aspects of IgA • Human but not mouse IgA comes in two isotypes, IgA1 and IgA2. Both isotypes are differently produced in various compartments and possess distinct properties. There is ongoing discussion to what extent IgA2 is generated by class switch recombination of IgA1 precursors in the gut. • Human serum IgA is almost exclusively monomeric whereas J chain-containing dimeric IgA is a distinguishing feature of mucosal sites. Thus, the structure of monomeric IgA in serum is fundamentally different from dimeric IgA in mucosal tissues. Only J chain-containing dimeric IgA is transported by the polymeric Ig receptors across mucosal epithelia. • Secretory IgA (SIgA) is not produced by B cells alone. SIgA is a hybrid molecule produced by the combined activity of Ig-producing plasma cells and of epithelial cells that provide the secretory component and mediate trans epithelial transport. SIgA is typically not present in serum but it is the dominant Ig isotype in tears, saliva, bile, colostrum, and the gut. Note: Secretory IgA shall be abbreviated by capital “S” to clearly distinguish from surface IgA (sIgA). IgA induction • In mice, T cell-dependent (TD) and T cell-independent (TI) pathways of IgA induction both contribute to IgA production. However, the relevance of TI and TD pathways of IgA induction in humans is unclear. The discussion of TD versus TI responses might need to consider both cognate and non-cognate T cell functions. • GALT comprises different structures and shows major variations between species. In humans, the dominating GALT structures are Peyer’s patches and isolated lymphoid follicles. Antigen binding • Fab-dependent binding of SIgA is conferred by the CDR region and adjacent motifs in the Fab arms. The specificity and affinity of this interaction can be modified by somatic hypermutation. Additionally, SIgA shows non-canonical binding. Non-canonical antigen binding can be conferred by glycans and might in particular contribute to target/coat the microbiota with IgA. • SIgA can bind to a range of intestinal antigens including self, enteropathogens, and toxins, and to the endogenous microbiota. SIgA binding to the microbiota shows a phenomenon referred to as cross-species reactivity, i.e., the binding of monoclonal IgA antibodies to different members of the microbiota. SIgA binding to the microbiota comprises canonical and non-canonical interactions.	

Another key aspect concerns the overall structure of IgA. IgA exists in two prevalent variants, as either monomeric or polymeric form. Monomeric IgA is present in human serum at about 2–3 mg/ml [6] and is thereby the second most abundant antibody isotype in human blood. Monomeric IgA (mIgA) contributes to immune regulation in blood but is less commonly implicated in the acute and pro-inflammatory immune response of acute infection [11]. It is mainly thought to be produced in the bone marrow, even though at least some of the IgA plasma cells resident in bone marrow seem to be generated by gut immune responses [12–15].

However, body-wide, the dominant form is polymeric IgA (pIgA), foremost dimeric IgA, although higher molecular weight types exist (see Box 1, caveats in understanding IgA, and Fig. 1). In dimeric IgA, two identical monomers are linked tail-to-tail through extensions of their Fc regions and an additional protein called J chain [16]. Production of J chain-containing dimeric IgA is a function of mucosal plasma cells and sets them apart from IgA-producing plasma cells in other compartments such as the spleen and bone marrow. Consistently, J chain-containing dimeric IgA, but not monomeric IgA, is selectively actively transported across mucosal epithelia. Note: In mucosal immunology, the term polymeric IgA is commonly used to refer to all higher molecular weight forms of IgA, including dimeric IgA. In the context of IgAN, some authors use the term polymeric IgA to refer to high molecular weight forms other than dimeric IgA.

## Secretory IgA in mucosal tissues

Secretion of polymeric IgA is mediated by the pIgR, a glycosylated transmembrane protein expressed by many secretory epithelial cells. pIgA produced by plasma cells and released into the tissue binds to pIgR on the basolateral side of the epithelial cell. Subsequently, the complex is internalized and transported in vesicles to the apical side of the epithelial cell. There, proteolytic cleavage releases the molecule whereby a small polypeptide provided by the pIgR remains covalently bound to the IgA dimer. The pIgR-derived fragment is known as the secretory component (SC) and the whole complex, including the IgA molecules, J chain and SC, is referred to as secretory IgA (SIgA) [16, 17]. Consistently, in mice lacking pIgR, dimeric IgA is largely lacking in mucosal secretions and accumulates in the serum [18].

pIgR-mediated secretion of SIgA is best described and most studied in the gut and, indeed, the gut harbors the largest population of mucosal plasma cells in the body [19]. However, in addition to the gut, SIgA is secreted into the lung by lung epithelial cells, into saliva by cells of the submandibular glands and into tears by cells of the lacrimal gland. Other notable sites of SIgA production are the lactating mammary glands that transport SIgA into milk to protect the newborn and cells of the liver that secrete SIgA into bile. In the healthy kidney, pIgR has been found to be expressed by scattered tubular cells and parietal epithelial cells and is upregulated in various kidney diseases upon kidney injury [20]. Not all of these compartments have been studied in depth and we have to assume that differences will exist between the regulatory circuits controlling SIgA secretion in different tissue. Nonetheless, available evidence suggests that pIgR activity and SIgA secretion is a regulated process and is modulated by various factors in the respective tissues.

## The origin of SIgA and pathways of IgA induction

IgA is a class-switched antibody and textbook knowledge would imply that class switch should occur in germinal centers and require T cell help. However, a seminal paper by Andrew MacPherson demonstrated that IgA is present in T cell-deficient mice [21]. This observation has laid the foundation for various studies trying to define T cell-dependent (TD) and T cell-independent (TI) pathways of IgA induction. While there is a general agreement considering the existence of TI IgA responses in mice, there is considerable debate with respect to the relevance of TI IgA in humans, the contribution of both pathways to overall IgA production, and the exact anatomical localization of the process. Indeed, the discussion of the relative prevalence of TI as compared to TD IgA responses and potential functional differences between TI and TD IgA is among the most controversial aspects of IgA biology (Box 1).

T cells play different roles in B cell responses. In the following paragraphs, we will first describe the role of B-T interactions in class switch recombination (CSR) and thereafter the role of T cells in somatic hypermutation (SHM). Finally, we will discuss recent observations suggesting that in the gut immune system, T cells might provide help to B cells in an atypical manner that does not rely on cognate interaction, an observation that may help to reconcile some of the contrasting ideas in the field.

### Class switch recombination (CSR)

Mature B cells express a B cell receptor (BCR) on their surface to recognize antigen. The BCR initially present on mature B cells is IgM, and these cells may also co-express IgD as BCR. The process of CSR allows B cells to use DNA recombination to change their Ig heavy chain region, i.e., to switch their Ig isotype from IgM to IgG, IgE, or IgA. CSR requires the enzyme activation-induced deaminase (AID), which introduces DNA lesions that are converted into double-strand breaks in the DNA and eventually leads to CSR (mechanism of CSR have been reviewed in [22]). In the mouse, the exons encoding for IgA constant regions are located furthest downstream in the Ig heavy chain locus. Thus, the genomic configuration of the gene locus dictates that in the mouse, switch to IgA is the final of all possible CSR events, since the exons encoding for all other isotypes heavy chains will be lost during the switch to IgA. In humans, the IgA2-encoding exons are furthest downstream in the locus, and thus, CSR to IgA2, like CSR to IgA in the mouse, does not allow for any subsequent CSR events. However, in humans, an IgA1-expressing B cell can potentially switch to IgA2, and indeed, IgA1 to IgA2 switch has been proposed [23]. Still, BCR sequencing of human gut plasma cells revealed only few clones shared between the IgA1- and IgA2-expressing gut B cells, suggesting that CSR from IgA1 to IgA2 is at best rare also in humans [24].

The questions of where IgA class switch occurs and how prevalent IgA1 to IgA2 CSR is in humans are frequently debated along with the contribution of TI versus TD responses. It is commonly thought that signalling through the BCR alone cannot induce CSR and that additional co-stimulatory signals are required. A major pathway to provide such co-stimulatory signals is CD40 engagement by CD40L-expressing activated T cells in the presence of cytokines such as interleukin-4 (IL-4). Thus, CD40-CD40L ligation forms a basis for CSR in TD IgA responses. However, CSR-promoting co-stimulation can also be provided by other pathways, including TI pathways such as Toll-like receptor (TLR) signalling. This is true not only for IgA responses but also for CSR to other Ig isotypes. Hence, CSR per se does not always require T cells.

### Somatic hyper mutation (SHM) and affinity maturation

Affinity maturation allows B cells to modify their immunoglobulin genes, potentially resulting in a BCR and antibodies with higher affinity for their cognate/specific antigen. The processes of affinity maturation and SHM are linked to TD responses in the germinal centers (GC). GC provide the microenvironment within secondary lymphoid tissues for antigen-specific B cells to proliferate and for SHM to introduce random variations in their antibody-encoding genes. T cells, specifically T follicular helper T cells in GC, provide survival signals to B cells, and competition for TD survival signals drives the selection of higher affinity B cell clones [25, 26]. GC are constitutively present in gut-associated lymphoid tissues (GALT, to be discussed in the next section), indicating ongoing stimulation and maturation of antigen-specific B cell responses in the gut immune system. Consistently, plasma cells in the human gut lamina propria are highly mutated [27]. These observations suggest that IgA-secreting gut plasma cells arise from B cells that underwent extensive affinity maturation [28] and TD responses in GC. However, presence of somatic mutations and GC does not formally exclude TI responses. Importantly, GC formation in GALT does not require BCR signalling and somatic mutations can occur in T cell-deficient settings [29]. Moreover, IgA coating intestinal microbiota is present in T cell-deficient mice. We proposed that TI IgA responses might be of particular importance in young individuals and thereby prominently show in experimental systems that frequently study young mice. In contrast TD IgA responses might dominate IgA induction in adults, in particular adult humans. However, this idea requires more in-depth validation and the importance of TI versus TD responses for IgA induction remains controversial (see [30]).

Some of the controversy might be resolved when considering the different roles that T cells can play during IgA responses. During textbook TD B cell responses, the interaction of T and B cells in GC relies on shared TCR and BCR specificity, respectively. I.e., T and B cells need to recognize antigen derived from the antigenic structure (note that this not only includes single molecules but also may comprise large structures such as viruses or bacteria). Such conventional cognate interactions drive B cell activation and maturation in GC. A particularly well-studied system to show such responses is the oral application of the strong mucosal adjuvant and antigen cholera toxin. However, mucosal IgA responses do not always rely on cognate T-B interaction. In mouse transgenic systems, GC formation could be observed in GALT even though T and B cell did not share antigen specificity [31]. This indicates that in GC of the GALT, T cells may deliver CD40-CD40L-dependent non-cognate signals. Similarly, mice lacking the SLAM-associated protein cannot form GC in peripheral lymph nodes and spleen but have GC in GALT [32]. Interestingly, such non-cognate and cognate T cell functions might localize to different microenvironments in GALT, and different molecular and cellular pathways may therefore govern T-B interactions in GC and pre-GC events that occur before B cells seed the GC [33]. We will discuss such differing T cell functions in more depth in the following in the context of the spatial-dynamic organization of IgA responses.

## Immune anatomy of IgA responses—Peyer’s patches

**Fig. 2:**
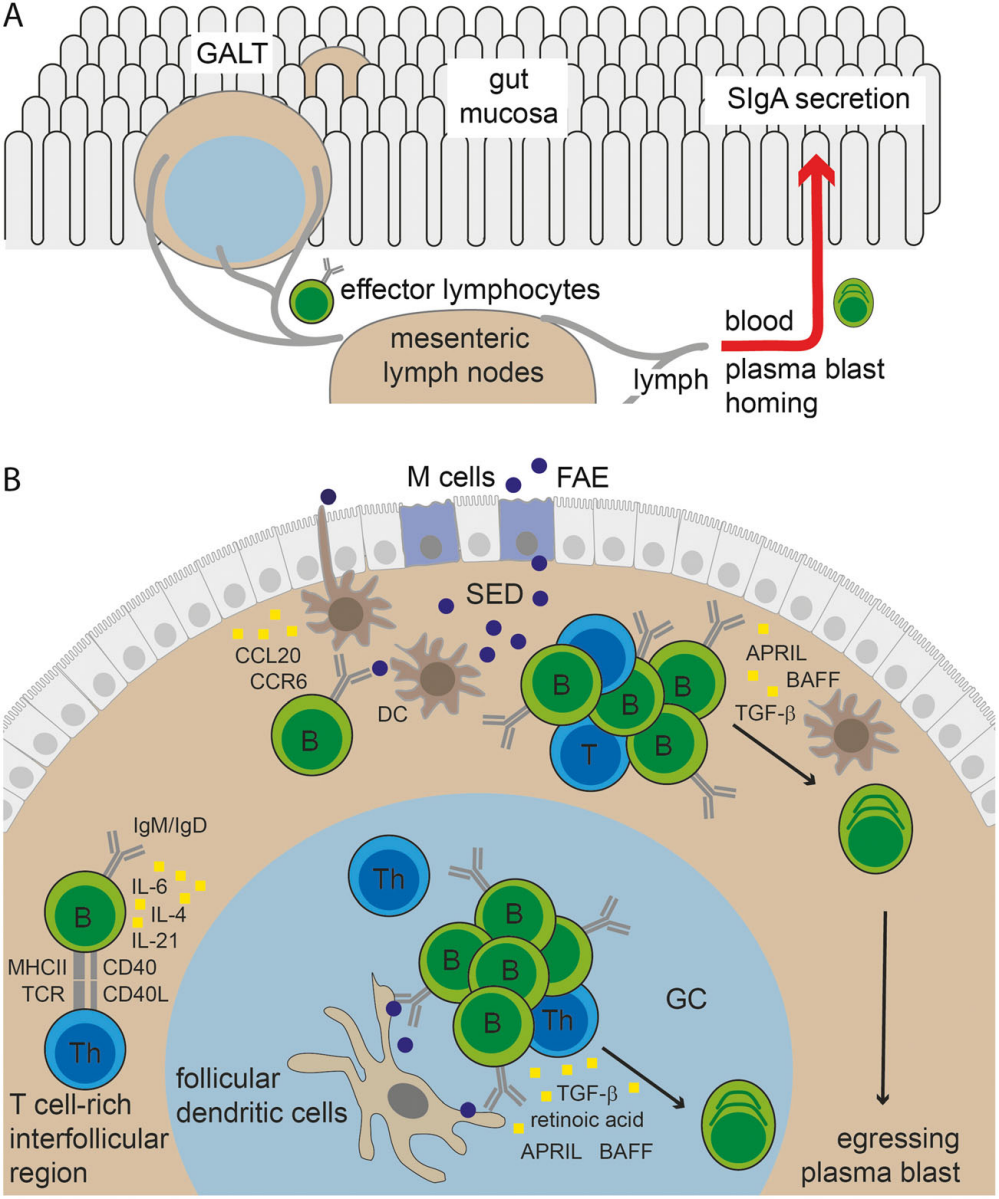
Immune anatomy of IgA responses. **A** Gut-associated lymphoid tissues (GALT) constitute the inductive sites of the gut immune system. In GALT, antigens are sampled and adaptive immune responses develop. Activated effector cells, including plasma blasts, egress from GALT, transit through the gut-draining mesenteric lymph nodes, and home to the gut mucosa via blood. In the mucosa, plasma cells produce dimeric IgA that is secreted by the gut epithelium as SIgA. **B** In Peyer’s patches, distinct regions enable key steps in IgA induction. Antigens (depicted as dark blue circles) are sampled from the gut lumen by M cells in FAE or myeloid cells that extend cellular protrusion to the gut lumen. The SED contains various myeloid cell populations including DC. B cells are attracted to the SED by the chemokine CCL20. B cells can interact with antigen-presenting cells in the SED directly inducing T cell-independent B cell proliferation. In parallel, B cells can differentiate into plasma blasts in a T cell-dependent response that does not rely on cognate interaction. Classical T cell-dependent IgA responses require T cell activation and differentiation into follicular T helper cells (Th) in the interfollicular region. Key factors in this interaction include TCR:MHCII and CD40:CD40L interaction besides various interleukins. Subsequently, B cells that receive T cell help differentiate into plasma blasts, memory B cells, or GC B cells. In GC, B cells undergo further maturation including somatic hypermutation in a T cell-dependent process that relies on cognate interaction and affinity-based selection. Various cytokines and other factors promote IgA induction, such as interleukins, TGF-β, APRIL, BAFF, and retinoic acid. Depiction of cytokines and other IgA switch promoting factors is by yellow squares. However, the exact cellular sources have not been established for all of these factors and the illustration shall not provide a complete summary of all available information

The gut immune system comprises effector and inductive sites (Fig. 2A). Effector sites are firstly the gut epithelium that mediates barrier functions and transepithelial IgA transport, and secondly, the underlying lamina propria that, in addition to other immune cell populations, also comprises the IgA-secreting plasma cells. Inductive sites comprise GALT and the gut-draining mesenteric lymph nodes. With respect to the induction of IgA responses, the main GALT compartments are Peyer’s patches (PP), the vermiform appendix, and the isolated lymphoid follicles (ILF). Mesenteric lymph nodes are thought to play a subordinate role in IgA responses.

In the human small intestine, PP and the vermiform appendix can be seen as large multifollicular lymphoid compartments. Both tissues develop during gestation and are bona fide secondary lymphoid organs. The exact number, size, and distribution of PP vary between individuals and with age but PP in human and mice are confined to the anti-mesenteric side of the small intestine, i.e., the large intestine does not have PP [34].

PP are considered important sites for IgA induction and maturation of IgA responses [9, 35]. This notion largely rests on experimental work in mice but is thought to be translatable to humans because of the similar PP organization and cellular composition in both species. By comparison to lymph nodes, PP contain comparably smaller populations of naïve and memory T cells. Instead, PP are dominated by numerous aggregated B cell follicles that typically possess GC that are situated close to the gut epithelium overlying the follicle. The region between PP follicles and the overlying epithelium is named the subepithelial dome (SED) and the epithelium shielding the PP SED from the gut lumen is known as follicle-associated epithelium (FAE) (Fig. 2B). The FAE is a specialized epithelium enabling direct antigen sampling from the gut lumen. PP lack afferent lymphatics and, instead, specialized epithelial cells, the microfold cells (M cells), in the FAE transcytose luminal antigen including microorganisms, prions, viruses, immune complexes, and inert particles [36, 37]. In mice lacking M cells, plasma cell development is delayed, and conversely, targeting antigen directly to M cells increases the efficacy of antigen-specific IgA responses [38]. These examples indicate that antigen sampling though M cells is a key step for the efficient induction of IgA responses against various intestinal antigens.

The exact cellular and molecular interactions that enable IgA induction in PP are not yet fully understood. PP enable canonical TD IgA responses relying on cognate interactions, CD40-CD40L interaction, and a GC cytokine environment that favors antibody production (Fig. 2B). Additionally, high concentrations of transforming growth factor-β, retinoic acid, and IL-21 promote IgA CSR in PP. Highly expanded B cell clones that show increased antigen affinity due to somatic hypermutation populate PP GC [39]. Somewhat counterintuitively, the presence of such highly selected PP GC was more prevalent in germ-free mice lacking a live microbiota as compared to colonized mice [39]. This hints at the importance of non-microbiota-derived antigens to select gut B cell responses. Irrespectively, there is a broad consent that GC in PP are key sites for IgA production and affinity maturation in both humans and mice [40].

In addition to conventional TD responses localized in GC, the SED has been suggested as a niche to foster interactions between B cells, T cells, and dendritic cells (DC). Positioning of B cells in the SED requires expression of the chemokine receptor CCR6 that binds its ligand CCL20 expressed by the FAE. In the SED, B cells interacted with DC that supported IgA CSR by integrin αvβ8-mediated activation of transforming growth factor-β (TGF-β) [41]. Moreover, DC in the SED can produce “a proliferation-inducing ligand” (APRIL), B cell-activating factor of the TNF family (BAFF), and TGF-β [42], cytokines that can promote IgA CSR [43]. Additionally, T cells resembling T follicular helper cells are localized in the SED and promote B cell expansion at a pre-GC stage (Fig. 2B). Interestingly, expansion of B cells in the SED did not entail clonal selection [33]. Thus, distinct steps of the IgA response might be organized in SED and GC of PP. In the SED, interaction with antigen, DC, and T cells can drive expansion of antigen-specific B cells and IgA CSR but does not imply B cell selection. High-affinity B cell clones that responded to antigen in the SED may enter into pre-existing GC to undergo TD B cell selection and affinity maturation. Consideration of various T cell functions, including cognate and non-cognate interactions, as well as a fine-grained characterization of the relevant microenvironments within PP are critical for an in-depth understanding of IgA responses that goes beyond the mere description as TD versus TI and GC-dependent versus GC-independent responses.

## Immune anatomy of IgA responses—isolated lymphoid follicles

Besides PP, isolated lymphoid follicles (ILF) are sites of IgA induction. Contrary to large multifollicular PP, ILF contain a single follicle and are typically less than 1 mm in diameter. Consequently, ILF cannot be seen when inspecting the human gut by naked eye but are numerous, present in both the small and large intestines and estimated to comprise about 30,000 structures [40, 44]. Therefore, the total number of B cells organized in ILF exceeds the number of B cells in PP. ILF come in different flavors. In humans, ILF have different anatomical locations and may either reside in mucosal lamina propria or localize mainly in the submucosa [24]. Like PP, ILF are characterized by B cells and GC, have a region that resembles the SED in PP, and possess an FAE that contains M cells and has an antigen sampling function. Thus, ILF show the anatomical hallmarks of PP and their cellular composition indicates that they function as adaptive immune-inductive sites for GC-based IgA induction [24].

ILF cells are typically co-isolated when cells are obtained from gut lamina propria. Such ILF-derived contaminants of lamina propria isolates prompted misleading interpretations of experimental data. For example, naïve B cells isolated from murine gut lamina propria are likely to derive from ILF and not from bona fide lamina propria. Similarly, the somewhat controversial suggestion that IgA CSR may occur locally in the lamina propria [45] might have come, at least in part, from ILF-derived B cells contaminating lamina propria isolates. While this problem has been tackled in mice, approaches to obtain human ILF for cell isolation and in-depth characterization were only recently developed [46].

While ILF and PP might both support the induction of IgA responses, in some settings, they may serve non-overlapping functions, e.g., a widespread availability of GALT in small and large intestines might enable the induction of regional IgA responses. Nascent plasma cells, also referred to as plasma blasts, egress from PP and ILF via efferent lymphatics, pass through mesenteric lymph nodes, and enter the blood circulation. From blood, GALT-derived plasma blasts specifically home back to the intestinal lamina propria by interaction of gut homing molecules, including the interaction of α4β7-integrin and its ligand mucosal addressin cell adhesion molecule-1 (MadCAM-1) in the gut. Interestingly, the homing of plasma blasts to the intestine shows regional specificity. In mice, IgA-expressing plasma blasts induced in small intestinal PP preferentially seed the small intestine, whereas blasts generated in the cecal patch, a structure in the murine cecum that resembles the small intestinal PP in its organization, show a comparably higher tendency to home to the colon [47]. Such selective homing of IgA-positive cells to small and large intestine is at least in part conferred by differential expression of the chemokine receptors CCR9 and CCR10 [47, 48] and of the colon homing receptor GPR15, which, along with α4β7-integrin, are key factors to guide immune cell migration into the gut. Indeed, B cells isolated from PPs and colonic ILF showed greater clonal overlap with plasma cells from the small or large intestinal LP, respectively [24]. This indicates that B cell responses in small intestinal PP might preferentially generate small intestinal plasma cells, whereas colonic ILF might preferentially yield plasma blasts with large intestinal homing properties. Yet, we are still lacking an in-depth understanding of how IgA responses in PP and different types of ILF are integrated and regulated to generate the intestinal plasma cell populations.

## Antigen specificity of SIgA

SIgA has different ways of binding antigens. The obvious binding modality is by canonical antigen binding conferred by the complementarity determining regions (CDR) and adjacent motifs in the Fab arms of the SIgA complex. Antigen specificity and affinity of Fab-dependent canonical binding can be modified by somatic hypermutation and affinity maturation. Additionally, non-canonical interactions between SIgA and antigen have been reported. Non-canonical binding relies on glycans decorating the hinge region, the J chain and the secretory component of SIgA (Fig. 1).

### Canonical antigen binding

Compared to systemic Ig responses, intestinal IgA responses are difficult to study. Oral antigen application results in tolerance induction rather than protective immunity [49] and repeated antigen encounters are required to stimulate detectable Ig responses. Thus, much of our understanding of intestinal IgA responses relies on comparably few experimental approaches. A particularly well-studied approach uses cholera toxin (CT). CT is an adenosine diphosphate (ADP)-ribosylating bacterial enterotoxin. Along with related toxins, such as *Escherichia coli* heat-labile toxins, it is the most potent mucosal adjuvant [50]. Oral exposure to CT induces TD responses in GC that generate CT-specific IgA responses that potently neutralize toxin effects in vivo.

Another informative approach to study IgA responses in vivo relied on the adoptive transfer of nitrophenol (NP)-specific transgenic B cells. In mice repeatedly challenged with NP-conjugated CT, NP-specific B cells acquired distinct high-affinity mutations [51]. Interestingly, different PP of an individual mouse contained clonally related B cells, indicating that the evolution of the B cell response seemed synchronized across different PP [51]. Consistently, we observed that, after depletion with a proteasome inhibitor, the intestinal IgA plasma cell population was rapidly reconstituted without major changes in the intestinal BCR repertoire [48]. Again, this observation hints at a recirculating B cell pool that re-enters GC in PP and potentially ILF and fuels the intestinal plasma cell pool. We speculated that re-entry of activated B cells might be a characteristic of intestinal B cell responses and that IgA responses might arise from the progressive maturation of B cell clones as the cells re-enter existing GC to undergo affinity maturation [19, 30].

### Non-canonical antigen binding

Non-canonical binding can be conferred by glycans and might in particular allow for SIgA binding to bacteria including the gut microbiota. Glycobiology of SIgA-microbiota interaction is only emerging as a field, arguably because of the technical challenges that come with the study of IgA glycosylation and glycan function. Glycobiology of IgA is a topic of major importance in IgAN, but also in this context, few original studies reported in-depth information on IgA glycosylation in the kidney of IgA patients.

Glycosylation of SIgA confers resistance to proteolytic cleavage but also confers antigen-binding capacity. A particularly instructive example of such non-canonical interaction between IgA and the microbiota is provided by the intestinal bacterium *Bacteroides thetaiotaomicron. B. thetaiotaomicron* expresses genes that allow binding to glycosylated SIgA of irrelevant Fab-dependent antigen specificity [52]. Thus, in this setting, the bacterium-IgA interaction is driven by the particular properties of *B. thetaiotaomicron*, rather than IgA specificity. Moreover, glycosylation of the secretory component in SIgA allowed binding to distinct intestinal bacteria [53, 54].

### SIgA binding to the microbiota

Thus, in particular, in the context of IgA-microbiota interactions, a careful study of both canonical and non-canonical interactions seems important. Fab-dependent canonical recognition of surface antigens may confer highly specific binding to distinct microorganisms but also efficient binding to diverse bacteria. Notably this may also include Fab-dependent binding to glycans. IgA responses to isolated glycans can arise from TI responses. However, in the gut immune system, glycans are not encountered as free molecules but in the context of whole bacteria. Thus, glycan-directed SIgA responses may arise from both TI and TD responses. In fact, in a collection of monoclonal antibodies generated from single human intestinal plasma blasts, we observed several highly mutated antibodies binding to a range of different members of the microbiota [55]. The very same antibodies showed highly specific binding to glycans (unpublished observation. J. Kabbert and O. Pabst). This observation is compatible with TD responses that would select for glycan-specific SIgA that can confer cross-species reactivity, i.e., IgA that can bind to different bacteria species.

## IgA in IgA nephropathy

IgAN is characterized by mesangial deposition of IgA-containing immune complexes. IgA deposits consistently re-occur in kidney-transplanted IgAN patients [56], whereas they often resolve after kidney transplantation from IgAN donors (with kidney IgA-immune complexes) to non-IgAN recipients [57]. This suggests that the primary defect in IgAN might not be in the kidney itself but rather in the IgA system.

There is an association of disturbance of the intestinal immune balance with IgAN episodes: Mucosal infections can trigger IgAN episode with macrohematuria [58, 59]. IgAN patients also show increased frequencies of *Helicobacter pylori*-specific IgA [60, 61], and there are hints that a gluten-free diet might potentially have an favorable effect [62] (yet randomized trials to confirm the role of gluten are missing). Finally, a recent randomized phase II trial using a targeted formulation of budesonide, designed to deliver the drug in the distal ileum, reduced proteinuria in IgAN patients [63]. Given these data and the key role of the gut immune system in the generation and regulation of IgA responses, an interest exists to explore whether perturbed gut IgA responses contribute to IgAN pathogenesis.

In many IgAN patients, IgA1 but not IgA2 serum levels are increased and elevated serum IgA1 in patients lacks the J chain and is mostly monomeric [64, 65]. However, elevated IgA levels per se do not trigger IgA deposition as seen in myeloma patients that show high IgA serum concentration but rarely develop IgAN [66].

IgA in IgAN patients shows an aberrant glycosylation, i.e., a reduced decoration of IgA-linked glycans with galactose. A lower proportion of β1,3 galactose linkages and sialylations are present and more terminal N-acetylgalactosamine (GAlNac) in the hinge of IgA becomes exposed and can be detected by binding of GalNac-specific lectins (Fig. 1 and [67]).

In vitro deglycosylation of IgA1 resulted in noncovalent self-aggregation and increased binding to components of the extracellular matrix [68]. Additionally, truncated glycans might act as neoantigens and become recognized by IgG and IgA antibodies, thus enhancing the formation of circulating immune complexes in the patients. Indeed, besides a higher proportion of undergalactosylated IgA (Gd-IgA), the blood of patients with IgAN contains increased numbers of mostly Gd-IgA1 and IgG-containing immune complexes in different constellations, e.g., IgA1-IgG or IgA1-IgA1, although the rare presence of IgA2 has also been reported [69–71]. Consistent with the properties of IgA in serum of IgAN patients, dedicated mass spectrometric studies confirmed the presence of undergalactosylated IgA in the glomerular deposits of IgAN patients [7, 72]. The today broadly accepted “multi-hit theory” emerged from these data, stating that (1) first Gd-IgA occurs in the circulation of patients, which (2) might lead to the formation of autoantibodies (IgG) against Gd-IgA1. Subsequently, (3) circulating immune complexes are formed, containing among others complexes of Gd-IgA1 and IgG. Finally, those become (4) deposited in the glomerular mesangium where they lead to complement activation, damage to mesangial cells and podocytes, and support renal fibrosis [73]. Besides, there are also reports of SIgA deposition in glomeruli of IgAN patients which showed a correlation with simultaneous mannose-binding lectin deposition, one of the recognition molecules of the lectin pathway of complement activation [74]. Although this theory is widely considered, many questions remain unanswered.

## Concluding remarks

The intestinal IgA system still holds many unknowns—as does the pathogenesis of IgAN. The basic defect underlying IgAN seems to be linked to changes in the IgA system (and not in the kidney). However, increased levels of IgA are not pathogenic per se and the nature of serum IgA and IgA-containing immune complexes in IgAN patients differs from the general intestinal IgA responses.

We recently named glycobiology as the potential “elephant in the room” in SIgA biology [30]. In the field of IgAN research, aberrant glycosylation has long been recognized and might drive alterations in the physicochemical and immunological properties of IgA. Aberrant glycosylation might also support the formation of IgA-containing immune complexes and IgA deposits in the kidney. In the future, mucosal immunologists may contribute to the understanding of IgAN by helping to identify the anatomical, cellular, and molecular pathways driving the generation of aberrant IgA responses in IgAN.

## Data Availability

Not applicable.
